# Automatic Nuclei Segmentation in H&E Stained Breast Cancer Histopathology Images

**DOI:** 10.1371/journal.pone.0070221

**Published:** 2013-07-29

**Authors:** Mitko Veta, Paul J. van Diest, Robert Kornegoor, André Huisman, Max A. Viergever, Josien P. W. Pluim

**Affiliations:** 1 Image Sciences Institute, University Medical Center Utrecht, Utrecht, The Netherlands; 2 Department of Pathology, University Medical Center Utrecht, Utrecht, The Netherlands; University of Campinas, Brazil

## Abstract

The introduction of fast digital slide scanners that provide whole slide images has led to a revival of interest in image analysis applications in pathology. Segmentation of cells and nuclei is an important first step towards automatic analysis of digitized microscopy images. We therefore developed an automated nuclei segmentation method that works with hematoxylin and eosin (H&E) stained breast cancer histopathology images, which represent regions of whole digital slides. The procedure can be divided into four main steps: 1) pre-processing with color unmixing and morphological operators, 2) marker-controlled watershed segmentation at multiple scales and with different markers, 3) post-processing for rejection of false regions and 4) merging of the results from multiple scales. The procedure was developed on a set of 21 breast cancer cases (subset A) and tested on a separate validation set of 18 cases (subset B). The evaluation was done in terms of both detection accuracy (sensitivity and positive predictive value) and segmentation accuracy (Dice coefficient). The mean estimated sensitivity for subset A was 0.875 (±0.092) and for subset B 0.853 (±0.077). The mean estimated positive predictive value was 0.904 (±0.075) and 0.886 (±0.069) for subsets A and B, respectively. For both subsets, the distribution of the Dice coefficients had a high peak around 0.9, with the vast majority of segmentations having values larger than 0.8.

## Introduction

Assessment of breast cancer prognosis from excision biopsy slides relies largely on the Bloom-Richardson grading system. It is based on semiquantitative scoring of the degree of tubule formation, nuclear pleomorphism, and mitotic rate, which has proven to be prognostically strong [1]. However, the scoring is done traditionally by visual examination through the microscope which has suboptimal reproducibility [2]. The use of automatic image analysis methods, which can provide reproducible quantitative parameters that describe the tumor tissue, has been suggested as a way to overcome this drawback [3]. Traditional image analysis of conventional glass slides was hampered by the selective approach due to limitations of the scanning equipment and the need for special stains [4]. The introduction of fast digital slide scanners that provide whole slide images has led to a revival of interest in image analysis applications in pathology. Optimal integration of such applications in pathology workflow necessitates using hematoxylin and eosin (H&E) stained slides since this is the standard staining protocol (the diagnostic process for each case always starts with staining the specimen with these dyes). Given the complexity and the diversity of the tissue appearance, the automatic analysis of H&E stained images can be very challenging.

Segmentation of cells and nuclei is an important first step towards automatic analysis of digitized microscopy images. Most of the developed cell and nuclei segmentation techniques revolve around active contours, watershed segmentation, pixel-wise clustering/classification or a combination of the above, supplemented by different pre-processing and post-processing steps and detection/localization schemes. Bamford and Lovell [5] used a dual active contour model for the task of segmenting cell nuclei from cytoplasm in conventional Papanicolaou stained cervical cell images. Cosatto et al. [6] detected candidate nuclei locations in breast histopathology images using the Hough transform and evolved an active contour around each point, rejecting malformed outlines with a trained classifier. They used the segmentation output for predicting nuclear pleomorphism scores, however the segmentation method by itself was not rigorously evaluated. Fatakdawala et al. [7] presented an expectation-maximization driven geodesic active contour with overlap resolution for segmentation of lymphocytes in breast cancer histopathology images. Ali et al. [8] presented an active contour model that integrates region, boundary and shape information, and showed that it can be used for nuclei, lymphocytes and gland segmentation in prostate and breast cancer biopsy images. Wienert et al. [9] proposed a method for nuclei detection and segmentation based on contour tracing and subsequent pruning of contours to retain the most probable ones. They evaluated the detection performance of the algorithm in a set of breast, liver, gastric mucosa and bone marrow images. Watershed segmentation is a method particularly suited for cell and nuclei segmentation [10,11]. The results of the classical watershed segmentation can be significantly improved by modifying the segmentation function (topographical relief) to contain regional minima only at specific locations that mark the objects of interest and the background. These markers can be obtained in a variety of ways and the process is usually application-dependent. Malpica et al. [12] examined the use of this technique in bone marrow and peripheral blood microscopy images. Marker-controlled watershed for segmentation and subsequent tracking of cells in time lapse microscopy was proposed by Yang et al. [13]. Huang et al. [14] described a method for segmentation of nuclei in hepatocellular carcinoma biopsy images based on marker-controlled watershed segmentation of initial contours followed by refinement with a snake model. Marker-controlled watershed, with markers produced by template matching, was also used by Kachouie et al. [15] for segmentation of mammalian cells in microscopy images. For a broader overview of the topic of image analysis in histopathology images we refer the reader to a recent review [16].

Although many nuclei/cell segmentation methods exist in the literature, they are usually closely related to the microscopy technique, tissue type, staining and target cell/nuclei types. Thus, they are not directly applicable to an arbitrary type of image. In this paper we present a marker-controlled watershed based technique for segmentation of cancer nuclei in H&E stained breast cancer histopathology images. In addition to the combination of the different processing steps, the novelty of the method lies in the multiscale approach to the pre-processing of the images and the marker extraction for the watershed segmentation, the use of multiple marker types and the relatively simple but effective merging of the segmentations produced at different scales and from multiple markers. This multiscale and multimarker approach yields much better results that simply performing segmentation at a single scale and with a single marker type. The method was evaluated with regard to both detection and segmentation accuracy on a set breast cancer images of diverse tissue appearance, and showed excellent results. In addition to the evaluation on our dataset, we evaluated our method on the dataset used in [9] and achieved comparable results.

## Materials and Methods

### Breast cancer cases

For this study a total of 39 slides from 38 patients from breast cancer excision biopsies were used. The slides were routinely prepared with the standard procedure consisting of formalin fixation and paraffin embedding of the tissue, followed by cutting of 3-5 µm thick sections and staining with H&E. The digitization of the complete slides was done using a ScanScope XT whole slide scanner (Aperio, Vista, CA, USA) at a magnification of ×40 (0.75 NA) and a resolution of 0.25 µm/pixel. JPEG2000 compression with a quality factor of at least 80 was used to reduce the storage requirements. With this compression type and quality, no visible compression artifacts were present in the digital slides. From each digital slide a representative region of approximately 1×1 mm was selected and marked by an experienced pathologist (PJvD) and graded for nuclear pleomorphism according to the Bloom-Richardson grading system (grade I, II or III ranging from good to poor prognosis). The regions of interest were selected using predefined guidelines that are also used when performing grading by pathologists. More precisely, only areas with high epithelial cellularity and preferably on the periphery of the tumor were selected. Regions with severe lymphocytic infiltration and necrosis were avoided, as well as regions with scanning artifacts and out-of-focus problems.

The regions were divided into two subsets. Subset A consisted of 21 slides and was used during the development of the segmentation procedure. These slides were selected by an experienced pathologist (PJvD) to represent the diversity in tissue appearance and to have an approximately balanced distribution of pleomorphism grades. Subset B consisted of 18 slides of consecutive patients collected from our Pathology Department archive based solely on the availability. The segmentation procedure was developed on subset A and validation was performed on subset B. All the experiments in this paper were performed on the selected representative regions from the digital slides.

### Ground truth segmentation

**Figure 1:**
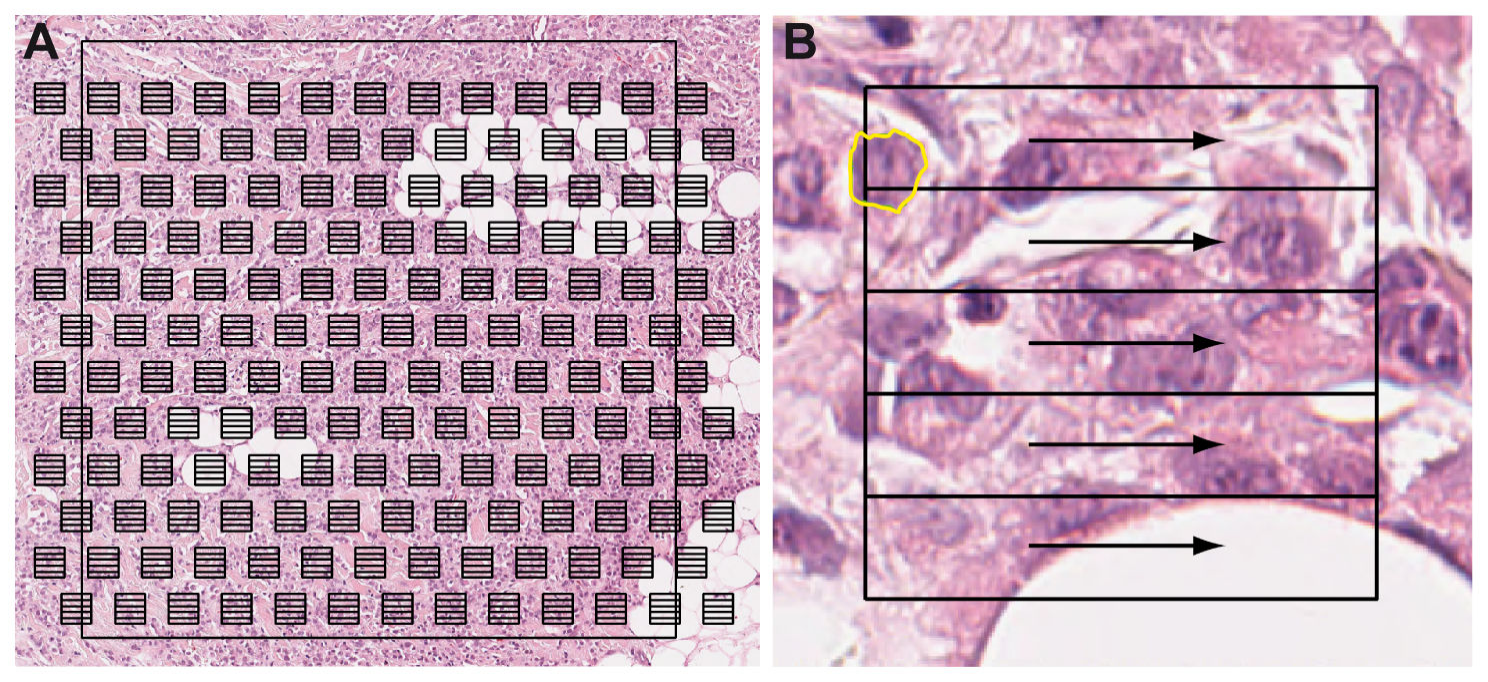
Systematic random sampling method used for manual nuclei segmentation. A) Systematic random sampling grid overlay on a representative region. B) One measurement frame from the sampling grid with a manually segmented nucleus (the arrows represent the scanning direction).

To set the gold standard, manual segmentation was performed in the marked regions on all 39 slides. Since each region contains several thousands of nuclei, manual segmentation of all nuclei was impractical and a systematic random sampling approach [17] was followed. This involved overlaying a grid of measurement frames over the marked region and segmenting one nucleus within each measurement frame (Figure 1.A). The grid was overlaid starting from an arbitrary location according to a distribution rule. The distribution rule depended on the area of the measurement frame and of the region, on the desired number of segmentations and on the estimated tumor area within the region (for more details see 17). Each measurement frame was subdivided into five rows. Scanning the rows from left to right, the first unscathed epithelial breast cancer nucleus with identifiable contours whose center of mass lied within the row was chosen for manual segmentation (Figure 1.B). Measurement frames of size 50×50 µm and a target of 100 nuclei per region were used. An expert (RK) performed one manual segmentation per measurement frame.

A summary of the dataset is presented in Table 1. We point out that in some cases the target number of 100 nuclei was not reached when too many of the sampling frames fell into non-tumor tissue, while in other cases this number was overreached. The sample size of 100 nuclei was chosen because it has been shown that this number of segmentations is sufficient to reliably estimate certain morphometric features such as the mean nuclear area [18]. At the resolution at which the digital slides were scanned, the average area of the manually segmented nuclei was approximately 900 pixels.

**Table 1:** Dataset summary.

	**Number of slides**	**Pleomorphism grade distribution (I, II and III)**	**Total number of manually segmented nuclei**	**Average number of manually segmented nuclei per slide**
**Subset A**	21	8; 8; 5	2191	104.3 (±12.2)
**Subset B**	18	1; 10; 7	2073	115.2 (±12.2)

Representative regions from Subset A were used for tuning of parameters during the development of the segmentation procedure. Representative regions from Subset B were used for an independent validation of the chosen parameters. From each slide, approximately 100 representative nuclei were manually segmented with systematic random sampling.

### Overview of the method

**Figure 2:**
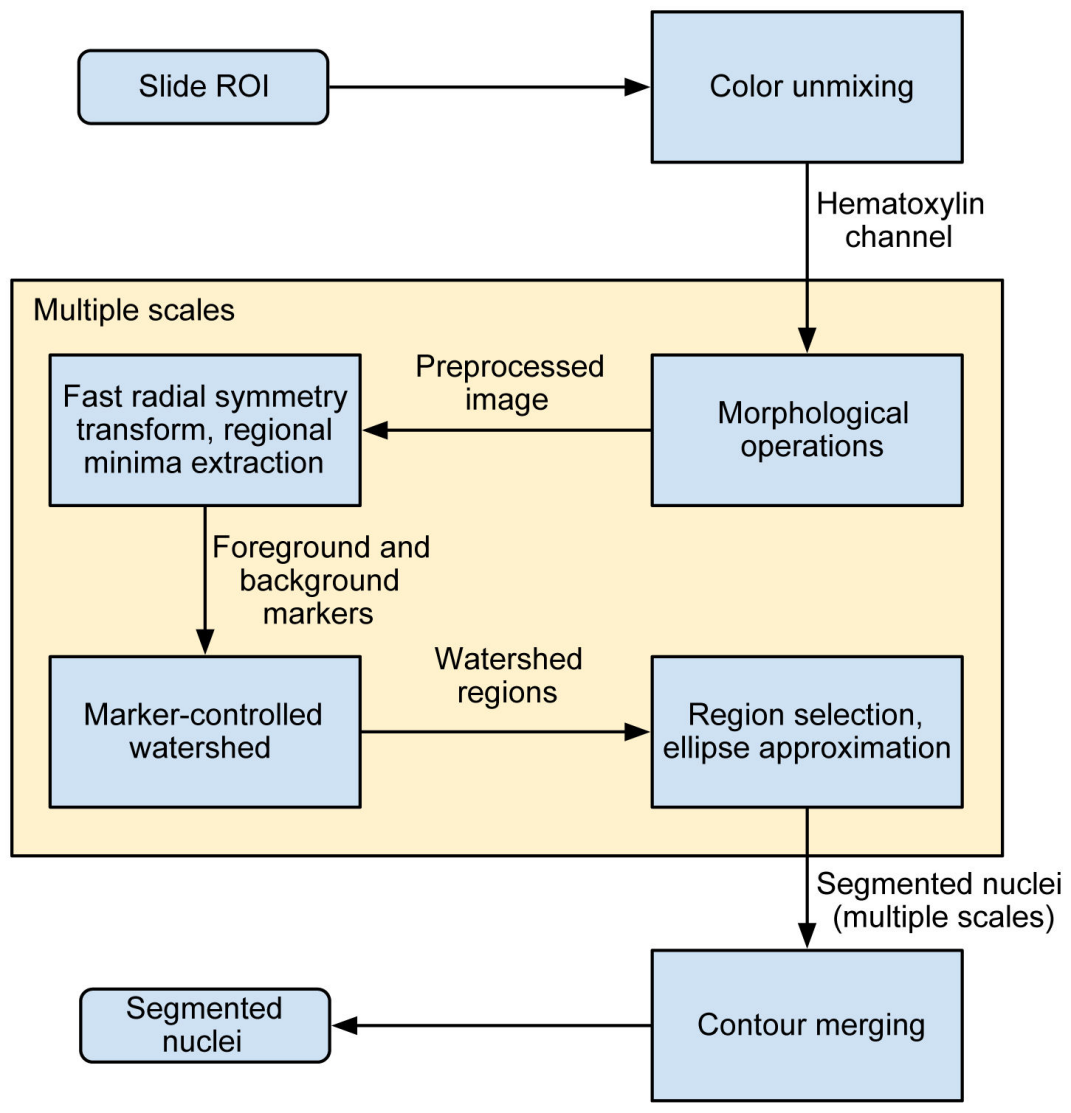
Schematic overview of the different steps in the automated image analysis method for nuclei segmentation.

**Figure 3:**
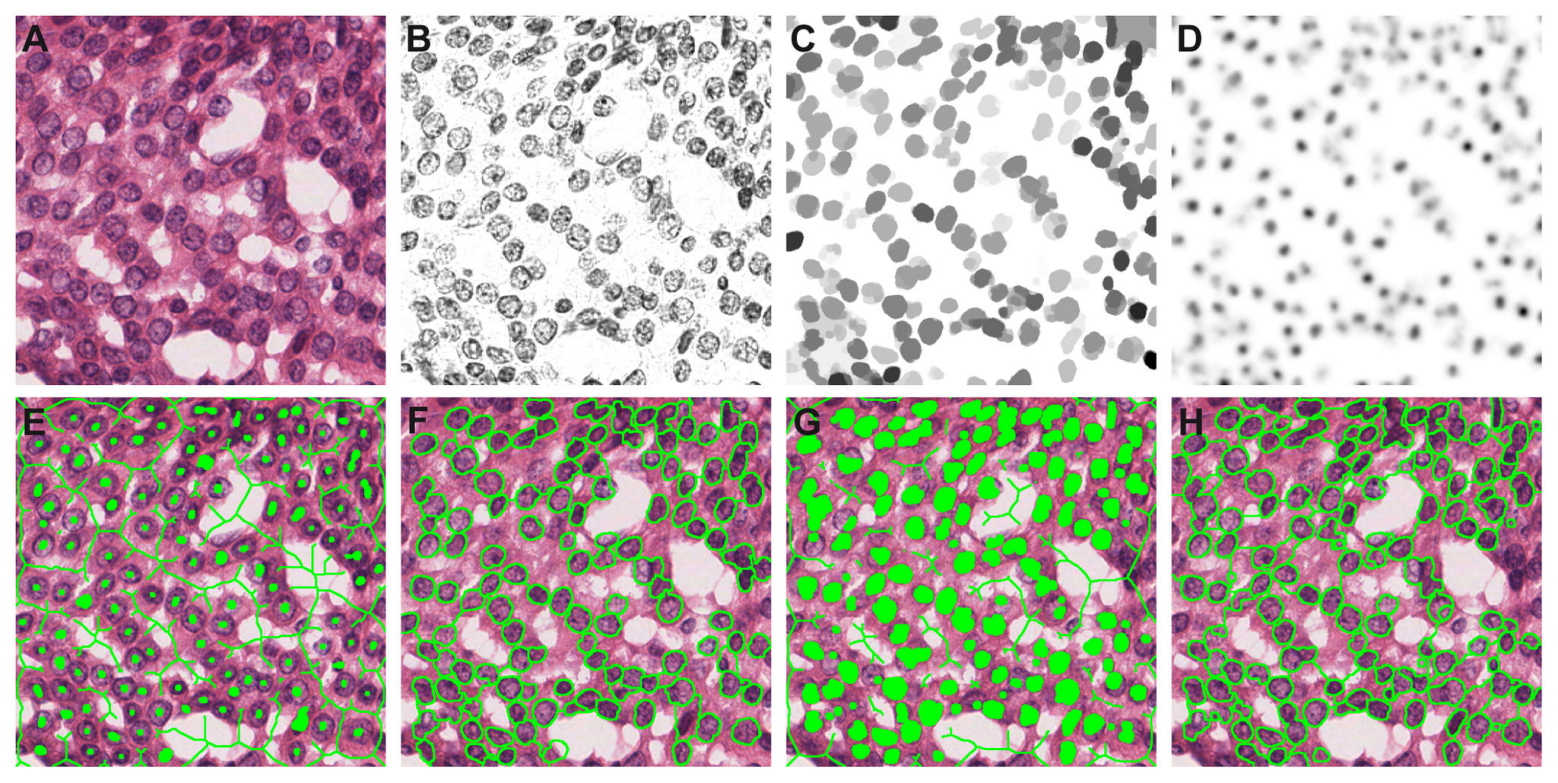
Marker imposition and watershed segmentation for nuclei segmentation. Prior to applying the FRST the image is preprocessed with color unmixing and morphological operations (*n* = 10). The set of radii for the FRST is *R* = (10, 11,…,20). Note: the markers and watershed ridges (given in green in the figure) were dilated by one pixel for better visualization. A) Original image. B) Hematoxylin channel. C) Pre-processed image (hematoxylin channel processed with series of morphological operations). D) Fast radial symmetry transform (FRST). E) FRST foreground and background markers. F) Watershed segmentation with FRST markers. G) Regional minima foreground and background markers. H) Watershed segmentation with regional minima markers.

A block-diagram with an overview of the proposed method is presented in Figure 2. This is an extension and improvement of our previously published nuclei segmentation method [19]. The entire procedure can be divided into four main steps: 1) pre-processing, 2) marker-controlled watershed segmentation, 3) post-processing and 4) merging of the results from multiple scales. The aim of the pre-processing is to remove irrelevant content while preserving the boundaries of the nuclei. The pre-processing starts with color unmixing for separation of the hematoxylin stain from the RGB image (the nuclei are dyed by this stain; Figure 3.B). The grayscale version of the hematoxylin image is then processed with a series of morphological operations in order to remove irrelevant structures (Figure 3.C). The core part of the procedure is the marker-controlled watershed segmentation. Two types of nuclear markers are used: markers extracted using an image transform that highlights structures of high radial symmetry (Figure 3.D–F) and regional minima of the pre-processed image (Figure 3.G-H). In the post-processing step, regions unlikely to represent nuclei are removed and the contours of the remaining regions are parameterized as ellipses. By varying the size of the structuring element in the pre-processing step, the segmentation procedure can be tuned to look for nuclei at different scales, allowing multiscale analysis. The segmentation results from the multiple scales and two marker types are then merged by resolving concurrent regions to give the final segmentation.

### Color unmixing

The first step is separation of the H&E stains with the color unmixing technique suggested in [20], which is a special case of true spectral unmixing techniques that work with multispectral cameras [21]. The technique uses the fact that the image formation process in bright field microscopy can be modeled by the Lambert-Beer law. Given that the images are captured by three detection channels (R, G and B) with known optical densities and the stain-specific absorption coefficients can be experimentally determined from single stain images, the concentrations of the two stains can be determined for each pixel location. These in turn can be used to obtain single stain images. Since the nuclei are stained with hematoxylin, the grayscale version of the hematoxylin single stain image is used in all subsequent processing. An example of color unmixing is presented in Figure 3.B.

### Morphological operations

The now separated hematoxylin image still contains spurious structures within the nuclei. These present obstacles for the marker extraction and segmentation and can be filtered out with a series of operations based on morphological grayscale reconstruction [22]. Opening by reconstruction removes unconnected bright objects that are smaller than the structuring element (SE). Similarly, closing by reconstruction removes unconnected dark objects smaller than the SE. Applying these two operators in sequence produces “flat” images and the amount of detail present can be controlled by the size of the SE. In the hematoxylin images, best results were obtained by first applying opening and then closing by reconstruction (both with a disk-shaped SE with radius *n*). The size of the SE, as defined by the radius *n*, should be chosen according to the size of the spurious structures which in turn is related to the size of the nuclei and the resolution of the image.

After application of these two operations the main contours of the nuclei often have an irregular shape and protrusions emanating from the edges hampering the segmentation result. To remedy this problem, additional morphological closing with a small SE is applied. This simplifies the shape of the object, eliminates small protrusions, disconnects “loosely” connected objects and does not significantly affect the location of the main contours. The SE for this operation is chosen to be a disk with half the radius of the one used for the opening and closing by reconstruction operators. An example of preprocessing with the series of morphological operations is shown in Figure 3.C.

It is difficult to set one parameter *n* that will work well across all images in our data set, or, in many instances, across different nuclei within one image. The optimal simplification factor is closely related to the size of the undesired structures that need to be removed (as all unconnected objects smaller than the SE will be removed). Employing a large SE oversimplifies the image, while using too small an SE does not always produce desirable results as many of the substructures within the large nuclei remain, affecting segmentation performance. This is why a multiscale approach was chosen – each image is preprocessed with SEs of different sizes and segmentation is performed at each scale. For the problem at hand, the range of SE radii is set to be *n*∈{10,11,…,18} pixels, which corresponds to the approximately expected range of minor semi-axes in breast cancer nuclei imaged at this magnification.

### Fast radial symmetry transform

The fast radial symmetry transform (FRST) [23] is a computationally efficient, non-iterative procedure that operates along the direction of the image gradient to infer centers of radial symmetry. This transform was originally developed for face detection tasks in computer vision, but was recently used in automatic analysis of follicular lymphoma [24,25] and bears similarity to other operators specifically designed for cell and nuclei segmentation [26]. A generalized version of this transform was used in [27] for segmentation of nuclei in breast cancer biopsy images.

The nuclear contours, in most cases, exhibited high radial symmetry making this transform suitable for their localization. To produce candidate nuclei locations, we use the orientation-based version of the transform, which discards gradient magnitude information and relies only on the orientation. This can be beneficial in the case of low contrast between the nuclei and the background. The FRST is computed for a set of radii *R* that reflects the size of the symmetric features that need to be detected. An example of the FRST applied to a morphologically pre-processed image is given in Figure 3.D.

### Marker imposition and segmentation

Given an input image preprocessed with the morphological operators at scale *n*, two marker-controlled watershed segmentations, each targeting a specific type of nuclei, are performed – one using FRST markers and one using regional minima markers. The FRST *S* is computed for the set of radii *R*∈{*n*,*n*+1,…,2n} pixels. This set of radii reflects the size of the nuclei that are reconstructed well in the preprocessed image. The FRST nuclei markers are extracted as the extended regional minima of *S*, with an empirically set height parameter *h* = 0.4. The extended regional minima of *S* are calculated as the regional minima of the *h*-minima transformation of S. The h-minima transform of S is given by:

$$S_{h}=\rho_{S}^{\varepsilon }\left(S+h\right)$$ (1)

with ρ the morphological grayscale reconstruction by erosion operator. This transform suppresses all minima in *S* whose depth is less than *h*.

For successful watershed segmentation the background also has to be marked. To achieve this, a naïve assumption that each detected foreground marker corresponds to a nucleus with maximal size (the largest radius in the set *R*) is made. In this way, provisional foreground (nuclei) and background maps can be formed. The morphological skeleton of the background map is used as a background marker.

After foreground and background markers have been obtained, the Sobel gradient magnitude image of the pre-processed image, which is used as a segmentation function for the watershed, is modified by imposing regional minima on the locations of the markers. In this way, only one watershed region per marker is obtained. Although the FRST markers are very successful in marking nuclei even in more complex situations like clustered nuclei, sometimes a proper marker is not produced in situations when the symmetry assumption is violated or in case of overly elongated nuclei. To address these situations, at each scale, an additional watershed segmentation is produced using the regional minima of the pre-processed image as markers as in [14]. The background markers are defined in the same way as for the FRST case. Figure 3 gives an example of marker-controlled watershed segmentation with FRST and regional minima markers. Frames 3E and G give the foreground and background markers from the FRST and the regional minima respectively, and corresponding results from the segmentation are given in Frames 3F and H.

### Post-processing

Many of the resulting watershed regions do not correspond to nuclei or represent erroneous segmentations (severe over- or under-segmentation, regions spilled into the background etc.). In the post-processing step we aim to remove those regions based on the following extracted features:

**Solidity** *s*: the ratio of the area of the object and of the convex hull of the object (the convex polygon with smallest area that contains the object). This value should be high for the nuclei regions since they are rarely concave. In our previous work [19] we have shown that this feature can be highly discriminative between correct and incorrect segmentations produced by marker-controlled watershed.

**Boundary saliency** *l*: the difference between the intensity level of the outside boundary and the intensity level of the inside boundary of the nucleus. The outside intensity level is taken as the median of the intensity values in a tight band around the segmented region. The inside intensity level is defined in an analogous way.

**Mass displacement** *d*: the distance between the centroid and the weighted centroid of the region (the pixel locations are weighted by the inverse intensity values) normalized by the smaller axis of the region. Low values of this feature imply near symmetric distribution of the intensity inside the nucleus region. In certain situations regions that do not correspond to correct segmentations have high mass displacement (regions spilled into the background, over-segmentations, under-segmentations etc.).

Although the problem of identifying the non-nuclei regions can be posed as a one- or two-class statistical classification task, we found that a simple rule-based rejection scheme is a much better and flexible solution. For each of the defined features a range of probable values is defined. If for a given region one of the features is outside of the probable range, the region is discarded. Additionally, regions that are too small (*area* < *n* ^2^π) or too large (*area* > 4*n* ^2^ π) for the scale at which they are segmented (as defined by *n*) were removed. Since the coarseness of the extracted contours depends on the scale at which they were extracted (smaller scales result in contours with finer details and vice versa), all the contours are standardized by approximating them with ellipses.

The ranges for the features were empirically determined and are as follows: *s*∈(0.875,1), *l*∈(20,255), *d*∈[0,0.08]. A qualitative analysis of the influence of the selected feature ranges is presented in Figure S1-S3 in the Supplementary Material. It can be observed that most of the segmentations outside of the excluded range correspond to false objects, and this effect is robust with respect to difference in tissue appearance.

### Merging results from multiple scales

The outputs from the multiple scales and the two types of markers often produce overlapping regions. For example, a nucleus might be properly segmented at a certain scale, but a substructure within the nucleus might be segmented at a higher scale, and/or oversegmentation containing another nucleus might be produced at a lower scale. Much more commonly, almost identical segmentations are produced at neighboring scales and/or with the two types of markers. These situations are resolved by identifying all overlaps and selecting the most probable regions according to a fitness value. For all pairs of regions (X_*i*_, X_*j*_) segmented in a given image *I* we define the following overlap measure:

$$OV\left({\text{X}}_{i},{\text{X}}_{j}\right)=\frac{\left|{\text{X}}_{i}\cap {\text{X}}_{j}\right|}{\text{min}\left(\left|{\text{X}}_{i}\right|,\left|{\text{X}}_{j}\right|\right)}$$ (2)

This measure has a maximum value of 1 when one of the regions is completely contained in the other one and a minimum value of 0 when the two regions do not intersect. Given this measure, the following adjacency matrix is defined:

$$A(i,j)=\{\begin{array}{cc} 1 & \text{if }OV({\text{X}}_{i},{\text{X}}_{j})>T_{h}\\ 0 & \text{otherwise} \end{array}$$ (3)

The threshold *T*_*h*_ defines when two regions are considered to be overlapping. All pairs of regions with a non-zero overlap measure smaller than this value are considered to be only “touching”. Each region is also assigned a fitness value *f* that is used for comparing concurrent regions and selecting the one that is most likely to represent a nucleus. The region overlaps are then resolved according to the following simple algorithm:

1. Find the region r with the maximum fitness value f (see below);
2. Mark r as accepted and reject all regions OVi that are adjacent to it;
3. Repeat steps 1. and 2. for the remaining regions until all are accepted or rejected.

The threshold *T*_*h*_ was chosen to be 0.2. This value allows small overlap of touching nuclei. Simply using the solidity of the region as a fitness value proved to give good results, although a linear combination of other features might be an alternative to consider.

### Evaluation

The automatic segmentations were compared with the manual segmentations obtained with systematic random sampling in the following way: if a manual segmentation was not intersected by an automatic segmentation with a Dice coefficient of at least 0.2, it was counted as a false negative (FN). Otherwise, it was counted as a true positive (TP). The Dice coefficient was taken as a measure of quality of the segmentation. The Dice coefficient is a measure of overlap between two regions, commonly used for evaluation of segmentation techniques. It is defined as:

$$D\left(X,Y\right)=2\frac{\left|X\cap Y\right|}{\left|X\right|+\left|Y\right|}$$ (4)

The reasoning behind a cut-off value of 0.2 was to avoid unsegmented nuclei that are “touched” by a neighboring segmentation to be counted as TP. The value of 0.2 is arbitrary, but it should be pointed out that in case of a lower value, more nuclei will be counted as TP at the cost of having more segmentations with very poor quality and vice versa.

To estimate the positive predictive value a subset of 100 automatically segmented nuclei from each slide was randomly generated. An expert (RJK) labeled all segmentations that did not correspond to epithelial nuclei, such as stroma, lymphocytes, “junk” particles etc.

For each representative region the sensitivity, positive predictive value and the median Dice coefficient were estimated. We refer to the sensitivity, positive predictive value and median Dice coefficient measures as estimates because they are based on an annotated subset of the entire population of nuclei in the images. Because of the asymmetric left-skewed distribution, the median of the Dice coefficient is a better measure of central tendency than the mean.

In addition to the evaluation on our dataset, we evaluated the proposed method on a publicly available dataset used in a recently published paper on nuclei detection and segmentation [9]. This dataset contains 36 histopathology images of breast, liver, gastric mucosa and bone marrow imaged at 20x magnification. The ground truth is provided as manually annotated nuclei centroids. We evaluated the detection performance on this data set in the same way as in [9], i.e. in terms of overall positive predictive value, sensitivity and conglomerate score (a score of the ability of the method to successfully separate conglomerates). For this experiment, no parameter values were adapted, except for the adjustment of the expected range of nuclei semi-axes, to account for the smaller magnification (*n*∈{5,6,…,9}).

## Results

**Figure 4:**
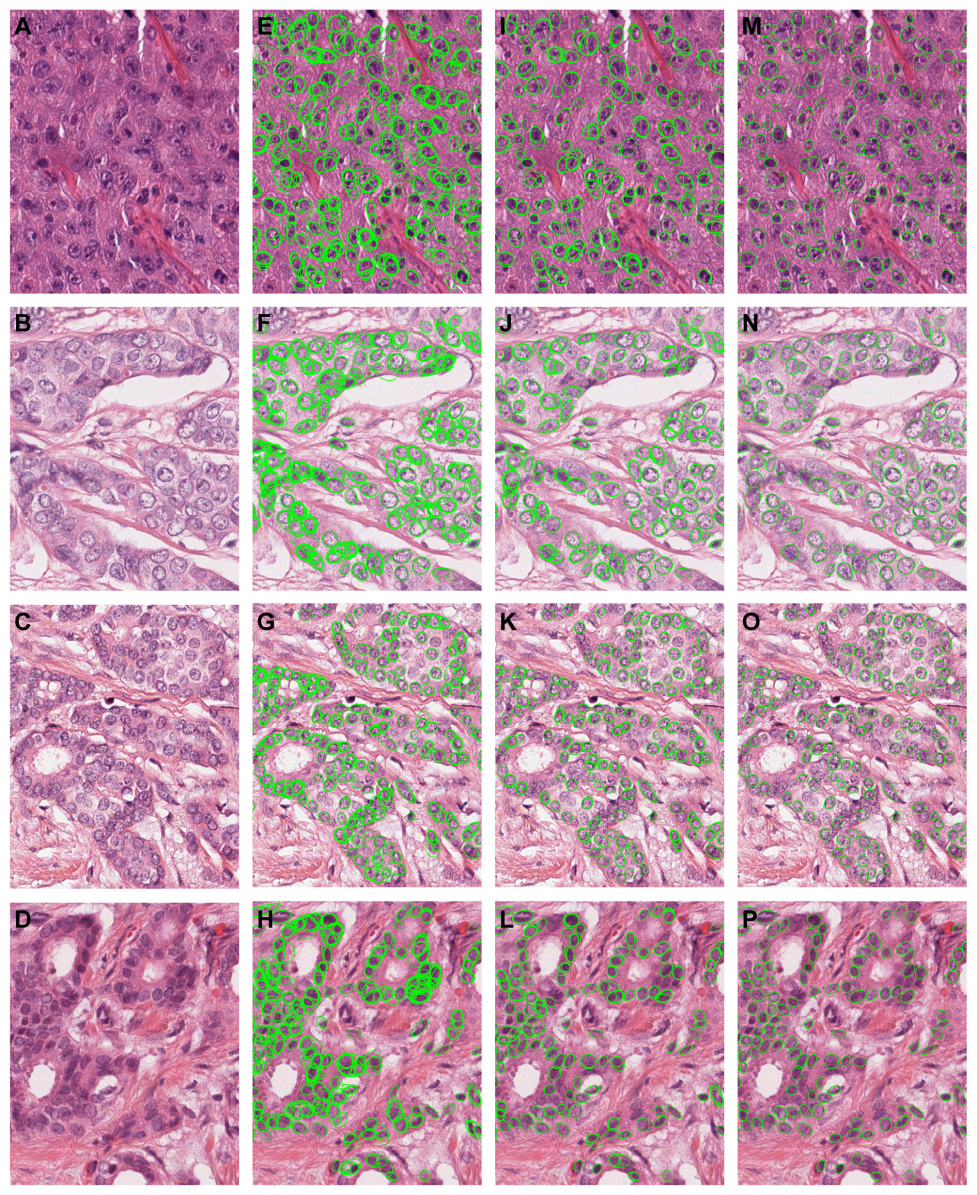
Examples of automated nuclei segmentation in breast cancer sections (all images are shown at the same scale; the nuclear pleomorphism grades are III, II, II and I respectively). A–D) Original images. E–H) Intermediate results prior to the rejection of spurious regions based on solidity, boundary salience and mass displacement. I–L) Intermediate results prior to the merging of contours from multiple scales. M–P) Final segmentation results.

Segmentation results for a few regions from our data set are given in Figure 4 for qualitative evaluation, along with the intermediate results prior to rejection of spurious contours and prior to the merging of concurrent regions. The four examples are chosen to represent tissue types with different appearance: large and small nuclei, nuclei organized into tubules, highly marginalized chromatin etc. In the same figure, the intermediate results prior to the rejection of false contours and merging of the contours from multiple scales are also shown. The visual examination shows overall good performance with a limited number of severe over- or under-segmentations. Also, it is apparent that a segmentation is produced for most of the nuclei in the image, with few contours corresponding to non-epithelial nuclei objects. The results from all the regions in our data set are available for download from: http://www.isi.uu.nl/People/Mitko/segmentation.html.

**Figure 5:**
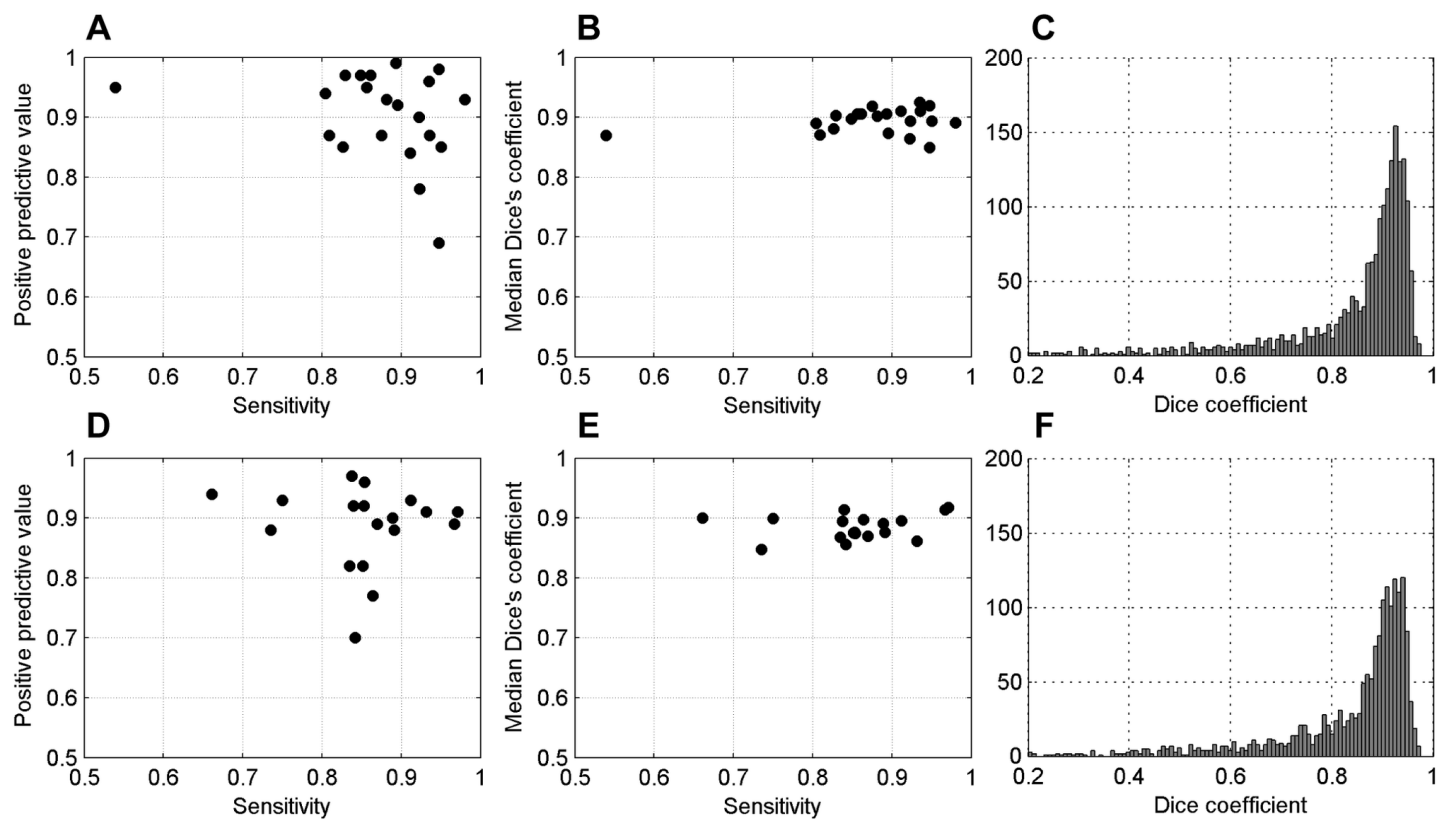
Plot of the performance measures. A–C) Performance measures referring to subset A. D–F) Performance measures referring to subset B.

The sensitivity, positive predictive value and median Dice coefficient for each case in subsets A and B are summarized in Figure 5. Note that subset A was used during the development of the algorithm and subset B is used as an independent validation set. The sensitivity was estimated as the percentage of manual segmentations that were matched to an automatic segmentation, as explained in the previous section. The positive predictive value was estimated as the percentage of the annotated automatic segmentations (100 per slide) marked as corresponding to an epithelial nucleus. The mean estimated sensitivity for subset A was 0.875 (±0.092) and for subset B 0.853 (±0.077). The mean estimated positive predictive value was 0.904 (±0.075) and 0.886 (±0.069) for subsets A and B, respectively. For both subsets, the distribution of the estimated Dice coefficients had a high peak around 0.9, with the vast majority of segmentations having values larger than 0.8.

The one outlier in terms of sensitivity in the first subset was due to the tissue being over-stained with eosin, which negatively affected the color unmixing procedure. The cases with low sensitivity in the second subset had a large proportion of nuclei that were not segmented due to their very small size (comparable to the size of lymphocytes). The outlying cases with low positive predictive value were either high grade cancer and/or had a large proportion of relatively large fibroblasts. In the high grade cancer cases, there were often many junk particles, usually of small size, that were picked up by the segmentation procedure. Although the scales for the segmentation were chosen so that most of the lymphocytes were not segmented, some were still included in the segmentation and they affected the positive predictive value negatively. Most of the segmentations had a high value of the Dice coefficient. The tail in the distribution of the Dice coefficients represents severe over- or under-segmentations (two or more nuclei segmented as one or a segmented sub-structure of a nucleus).

Specifying wider ranges of probable feature values during the post-processing will result in higher sensitivity but at the cost of decreasing the positive predictive value, and vice versa. Figure S1-S3 illustrate that the solidity feature is the most discriminative between true and false segmentations. This is because highly convex segmented regions are unlikely to occur by chance, and the convex regions that do occur correspond to correctly segmented nuclei in the vast majority of cases. This motivated the use of this feature as a fitness value during the region merging process.

A comparison of our multiscale method to the same method on only a single scale and with a single marker (*n* = 12 and FRST markers were chosen as best performing) showed that the sensitivity of the multiscale method on the validation set was significantly higher (0.853 compared to 0.579 on average). This exemplifies the added value of our multiscale approach.

Our method generalized well when used to detect and segment nuclei in a diverse set of histopathology images, including breast, liver, gastric mucosa and bone marrow tissues. We achieved an overall positive predictive value of 0.904, sensitivity of 0.833 and a conglomerate score of 0.989 which is comparable to the results of the method presented in [9] (0.908, 0.859 and 0.958 respectively).

**Figure 6:**
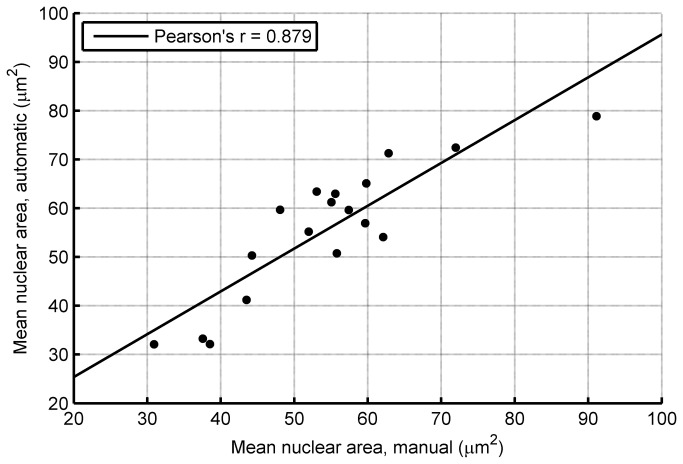
Scatter plot of the mean nuclear area as calculated by manual and automatic segmentation of nuclei.

One of the potential uses of an automatic nuclei segmentation method is to extract prognostically meaningful morphometric parameters. As an example, we show that the proposed nuclei segmentation technique can be used to reliably estimate the mean nuclear are (MNA) from the representative regions. The area of all segmented nuclei was calculated and then averaged for each representative region to produce the MNA. We trained a linear regression on the training set to correct for the systematic underestimation of the MNA. We observed that the main reason for this systematic underestimation is that the “junk particles” that are segmented are typically several times smaller than that of the large epithelial nuclei. In addition, undersegmentation of large nuclei is more common that oversegmentation of small nuclei. The learned linear regression was used to correct the MNA estimates of the cases in the validation sets. The results are presented in the form of a scatter plot in Figure 6. It can be observed that there is good correspondence between the two measurements and that there is no noticeable systematic bias.

## Discussion and Conclusions

This study set out to develop a segmentation method for breast cancer nuclei that works on H&E stained breast cancer histopathology images. The evaluation revealed that the proposed method has good performance in both detection and segmentation accuracy. The evaluation was done on two subsets of images, one of which was used for parameter tuning and the other for validation. The segmentation results were slightly worse for the validation subset, probably due to the fact that this data set contained more cases with high grade cancer that are generally more difficult to segment. Nevertheless, the results on this validation set provide a good idea of the performance of the algorithm in real life scenarios.

We did not perform standardization of the tissue appearance [28], in as much as the techniques we used aim for robustness with respect to variation in the preparation of the samples that is within the “nominal range”. However, it should be noted that very poor sample preparation (such as very thick sections, overstraining, poorly fixed tissue etc.) or poor digitization (failed autofocusing, stitching artifacts etc.) can adversely affect the segmentation technique. Still, these problems rarely occur and can be remedied with a stricter quality control during the tissue preparation and slide scanning.

One possible point of improvement of our segmentation technique may be the inclusion of a pre-segmentation step that divides the tissue into epithelial and stromal regions. This would help to eliminate some of the false positives that arise in the stromal areas. Another improvement would be the use of a dedicated lymphocyte segmentation/detection procedure, as presented in [7].

In our current work, we decided to concentrate on nuclear size features and nuclear architecture, because these are more robust with respect to the tissue preparation and staining processes compared with nuclear shape and chromatin texture features. For this purpose, elliptical approximations of the contours were sufficient. However, this approximation is a drawback when certain morphometric shape features need to be calculated. If computation of shape features is required, our segmentation algorithm can be extended to include an additional step of refining the contours.

The implementation of the method was done in MATLAB. The segmentation procedure for one image of size 1000×1000 pixels takes approximately 90 seconds on a PC with an Intel Core2Quad Q9500 processor. We note that this is only an experimental implementation, with processing times too slow for full slide segmentation, but further speed improvements are possible. In addition to this, tissue sampling methods [29,30], and/or supervised extraction of relevant regions of interest [31,32] can be used in order to reduce the number of regions from the full slide that need to be processed, while still providing a relevant result.

In another recent study [33] we have shown that the mean nuclear area (MNA) measurement extracted with the method presented in this paper is a relevant prognostic marker in a cohort of 101 male breast cancer patients, outperforming the traditional nuclear pleomorphism score. Development of other prognostic markers, derived for example from analysis of the nuclear texture or architecture of the tissue is also a possibility. This analysis can potentially be done on whole slide images, which opens the possibility for integration into the workflow of routine pathology practice. Segmentation of nuclei can also be used, in a bottom-up manner, to locate the tumor regions within the slide or to assess the degree of tubule formation.

In conclusion, we have presented an accurate technique for automated segmentation of nuclei in images derived from digital slides of H&E stained breast cancer sections. The technique was evaluated on a number of representative regions and showed good performance in terms of detection and segmentation accuracy. This technique can be used to estimate prognostically relevant quantitative features such as MNA for breast cancer grading.

## Supporting Information

Figure S1 Contours with increasing values for the solidity feature. The range of values from left to right: *s*∈(0,0.5),*s*∈(0.5,0.75),*s*∈(0.75,0.875),*s*∈(0,875,0.9375),*s*∈(0.9375,1).
(TIF)

Figure S2 Contours with increasing values for the boundary saliency feature. The range of values from left to right: *l*∈(-255,0), *l*∈(0,10),*l*∈(10,20),*l*∈(20,40),*l*∈(40,255).
(TIF)

Figure S3 Contours with increasing values for mass displacement feature. The range of values from left to right: *d*∈[0,0.02], *d*∈[0.02,0.04],*d*∈[0,0.08],*d*∈[0.08,0.16],*d*∈[0.16,1].
(TIF)
