# Supplementary figures and images for: Automatic Nuclei Segmentation in H&E Stained Breast Cancer Histopathology Images
Source: PLoS One. 2013 Jul 29;8(7):e70221. doi: 10.1371/journal.pone.0070221 (PMC3726421; doi:10.1371/journal.pone.0070221)

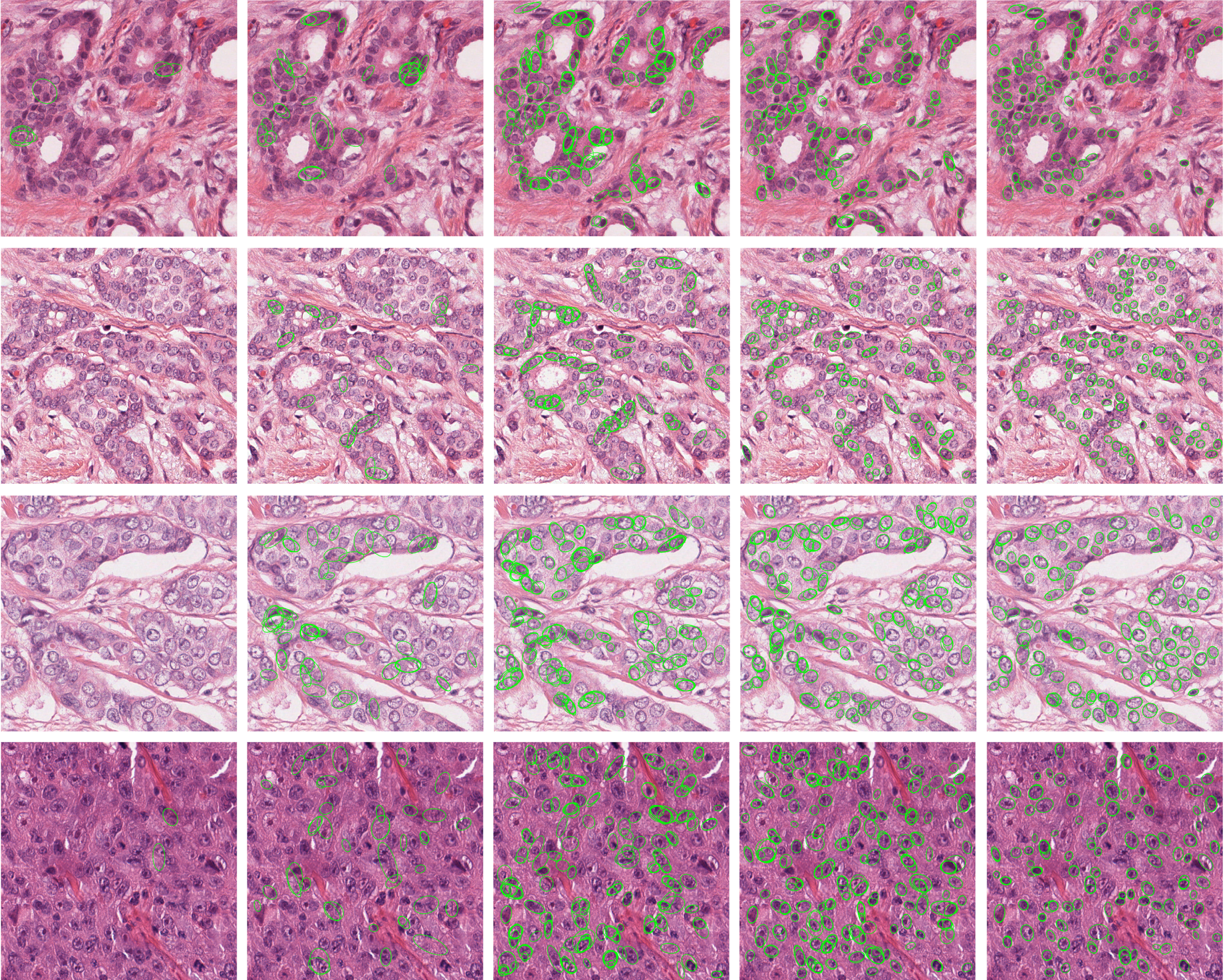

Supplement: Figure S1 — The range of values from left to right: s∈(0,0.5),s∈(0.5,0.75),s∈(0.75,0.875),s∈(0,875,0.9375),s∈(0.9375,1). (TIF) [file pone.0070221.s001.tif]

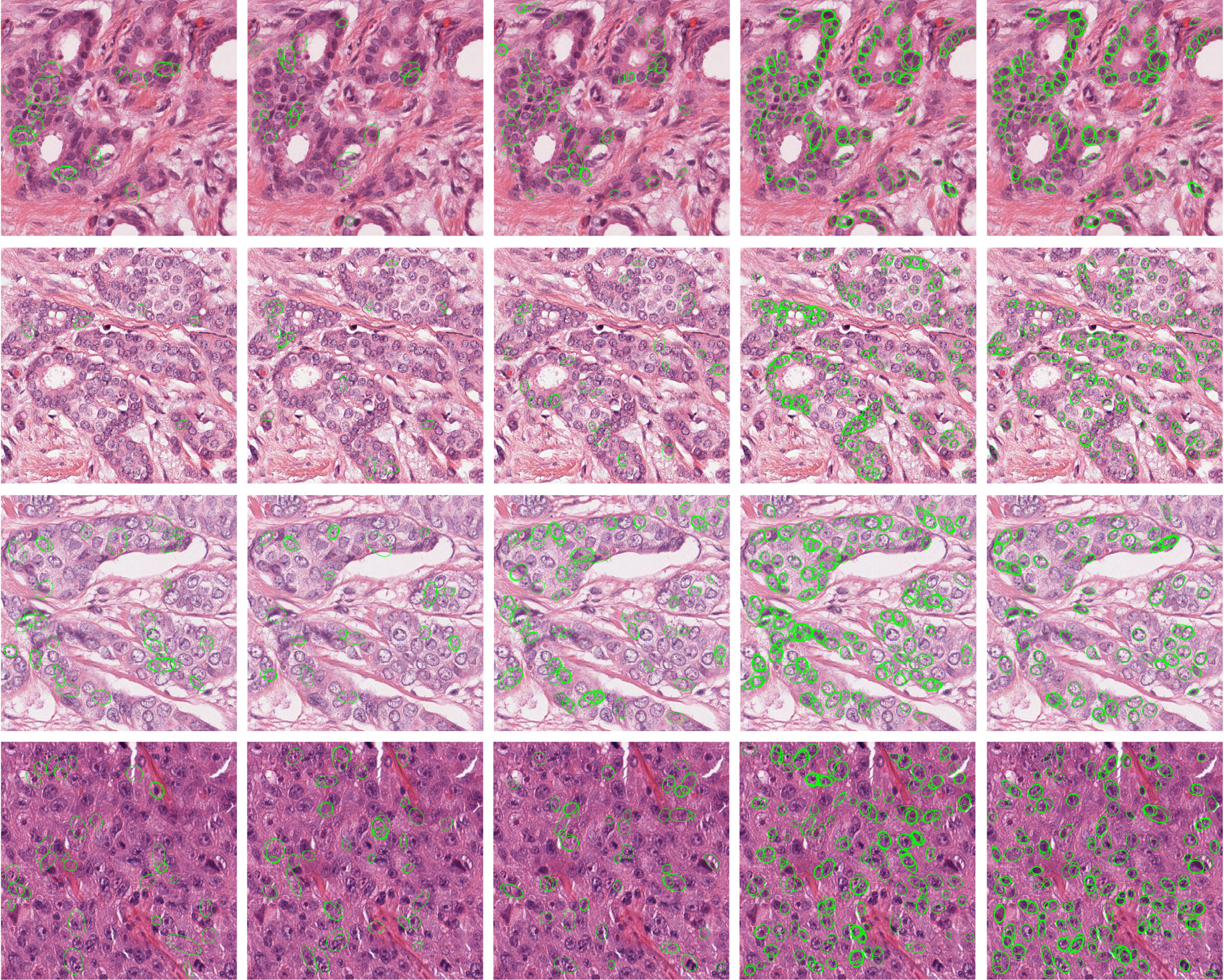

Supplement: Figure S2 — The range of values from left to right: l∈(-255,0), l∈(0,10),l∈(10,20),l∈(20,40),l∈(40,255). (TIF) [file pone.0070221.s002.tif]

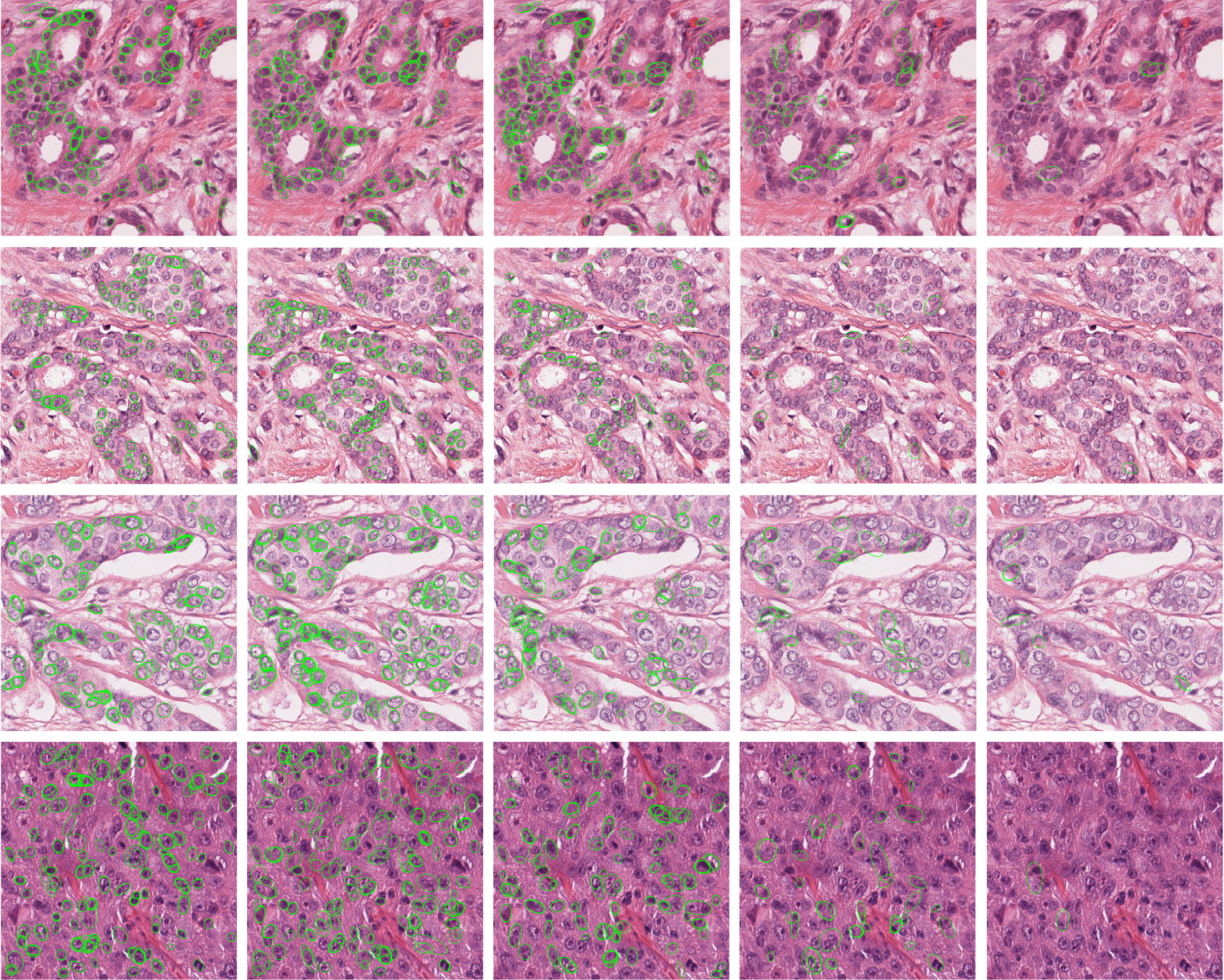

Supplement: Figure S3 — The range of values from left to right: d∈[0,0.02], d∈[0.02,0.04],d∈[0,0.08],d∈[0.08,0.16],d∈[0.16,1]. (TIF) [file pone.0070221.s003.tif]
